# Simulating the effect of school closure during COVID-19 outbreaks in Ontario, Canada

**DOI:** 10.1186/s12916-020-01705-8

**Published:** 2020-07-24

**Authors:** Elaheh Abdollahi, Margaret Haworth-Brockman, Yoav Keynan, Joanne M. Langley, Seyed M. Moghadas

**Affiliations:** 1 Agent-Based Modelling Laboratory, York University, Toronto, ON M3J 1P3 Canada; 2 National Collaborating Centre for Infectious Diseases, Rady Faculty of Health Sciences, University of Manitoba, Winnipeg, MB R3E 0T5 Canada; 3 Department of Community Health Sciences, and Max Rady College of Medicine, University of Manitoba, Winnipeg, MB R3E 0T5 Canada; 4 Department of Medical Microbiology, Max Rady College of Medicine, University of Manitoba, Winnipeg, MB R3E 0T5 Canada; 5 Canadian Center for Vaccinology, Dalhousie University, IWK Health Centre and Nova Scotia Health Authority, Halifax, NS B3K 6R8 Canada

**Keywords:** COVID-19, School closure, Self-isolation, Social distancing, Pandemic, Simulation

## Abstract

**Background:**

The province of Ontario, Canada, has instituted indefinite school closures (SC) as well as other social distancing measures to mitigate the impact of the novel coronavirus disease 2019 (COVID-19) pandemic. We sought to evaluate the effect of SC on reducing attack rate and the need for critical care during COVID-19 outbreaks, while considering scenarios with concurrent implementation of self-isolation (SI) of symptomatic cases.

**Methods:**

We developed an age-structured agent-based simulation model and parameterized it with the demographics of Ontario stratified by age and the latest estimates of COVID-19 epidemiologic characteristics. Disease transmission was simulated within and between different age groups by considering inter- and intra-group contact patterns. The effect of SC of varying durations on the overall attack rate, magnitude and peak time of the outbreak, and requirement for intensive care unit (ICU) admission in the population was estimated. Secondly, the effect of concurrent community-based voluntary SI of symptomatic COVID-19 cases was assessed.

**Results:**

SC reduced attack rates in the range of 7.2–12.7% when the duration of SC increased from 3 to 16 weeks, when contacts among school children were restricted by 60–80%, and in the absence of SI by mildly symptomatic persons. Depending on the scenario, the overall reduction in ICU admissions attributed to SC throughout the outbreak ranged from 3.3 to 6.7%. When SI of mildly symptomatic persons was included and practiced by 20%, the reduction of attack rate and ICU admissions exceeded 6.3% and 9.1% (on average), respectively, in the corresponding scenarios.

**Conclusion:**

Our results indicate that SC may have limited impact on reducing the burden of COVID-19 without measures to interrupt the chain of transmission during both pre-symptomatic and symptomatic stages. While highlighting the importance of SI, our findings indicate the need for better understanding of the epidemiologic characteristics of emerging diseases on the effectiveness of social distancing measures.

## Background

The global spread of the novel coronavirus (SARS-CoV-2) has triggered the implementation of community-based public health measures around the world [1, 2], including school closures (SC) [3], to mitigate the impact of the COVID-19 pandemic. The evidence that SC reduces disease spread in the community comes mostly from experience with influenza virus [4, 5]. Children are thought to play an important role in influenza transmission through close contact with classmates, friends, teachers, caregivers, and family. Furthermore, they have a reduced ability to implement hygiene and may have higher viral shedding for a longer period than adults [6]. However, evidence supporting SC to reduce infectious disease transmission in the wider community is uneven [7–10] and the epidemiology of influenza virus and SARS-CoV-2 may differ in important ways. For example, the incubation period of COVID-19 is estimated to have an average of 5.2 days [11, 12], compared to 1.5 days estimated for influenza A viruses [13]. Furthermore, the reproduction number of COVID-19 exceeds 2 in most settings, considerably higher than estimates for influenza in the range 1.2–1.8 [14]. These factors may significantly influence the effect of SC during COVID-19 pandemic.

At the time of writing, the province of Ontario has instituted indefinite SC [15] as well as other social distancing measures. SC is disruptive to families and society with measured and unmeasured direct and indirect costs [16–19]. Determining the optimal duration of SC may depend on emerging local epidemiologic data and learning from the experience of other countries that have implemented closures earlier in this pandemic [3, 8, 20]. The effect of SC on COVID-19 epidemiology is likely to be affected by other measures such as self-isolation (SI). It is therefore imperative to evaluate the potential outcomes of this public health strategy, along with other social distancing measures, to inform decisions on the continuation of SC during current outbreaks or future waves of COVID-19.

In this study, we developed and parameterized a computational model with demographics of Ontario, Canada [21], and the latest estimates of COVID-19 characteristics [11, 22], to simulate disease spread. We varied the length of SC under a range of scenarios for voluntary SI of symptomatic infected individuals who reduce their contacts within the community by staying at home. Compared to SI, our results indicate that SC may have limited impact on the overall attack rate (i.e., the proportion of the population infected throughout the outbreak) and the subsequent need for critical care of COVID-19 patients. We explicate that the longer incubation [11, 22] and pre-symptomatic [23] periods and larger reproduction numbers of COVID-19 [11, 24, 25], compared to influenza, are key epidemiologic attributes determining low effects of SC alone during COVID-19 outbreaks.

## Methods

### Setting

The province of Ontario is home to over 38% of Canada’s population (14.5 million) and is the second largest province by land mass (1,076,395 km^2^). Children attend publicly funded community and private schools from kindergarten to grade 12, beginning at age 4, from September to June each year.

### Model structure

We developed an age-structured agent-based simulation model and considered various individual compartments, including susceptible; infected and incubating (not yet infectious); asymptomatic (infectious), pre-symptomatic (infectious), and symptomatic (infectious) with either mild, severe, or critical illness; recovered; and dead. We stratified the population into five age groups of 0–4, 5–19, 20–49, 50–64, and 65+ years based on the latest Canadian census demographic data for the province of Ontario [21]. Disease transmission was simulated within and between these age groups by considering an empirically determined contact network [26, 27]. Daily inter- and intra-group number of contacts for each individual was sampled from an age-specific negative-binomial distribution, based on a contact matrix for urban and densely populated regions [26, 28]. When SC was implemented, the daily number of contacts was reduced in the model by either 60% or 80% among individuals aged 5–19 years [4]. The contact patterns between school children and other age groups remained unaltered [4, 29].

### Disease dynamics

Disease transmission was implemented probabilistically for contacts between susceptible and infectious individuals. If infection occurred, we considered two distinct paths, either symptomatic or asymptomatic, for the entire course of disease (Additional file 1: Fig. S1). An age-dependent proportion of infected individuals was assumed to develop symptoms after an average incubation period of 5.2 days estimated for COVID-19 [11, 12]. The period was sampled from a Log-Normal distribution [11, 12] and included a highly infectious pre-symptomatic stage prior to the onset of symptoms (Additional file 1: Table S1) [11, 12, 22–24, 30–34]. The duration of pre-symptomatic stage was sampled from a Gamma distribution with mean of 2.3 days [23]. Symptomatic cases remained infectious from the start of symptoms until recovery with a duration of disease communicability which was sampled from a Gamma distribution with mean 3.2 days [31]. Symptomatic cases had an age-dependent probability of developing mild, severe, or critical illness [35]. The remaining proportion of infected individuals remained asymptomatic after the incubation period until recovery. For asymptomatic cases, the infectiousness started during the incubation period, and its duration was sampled from a Gamma distribution with mean of 5 days [31]. Since infectiousness is estimated to reach the highest level 0.7 days (on average) before symptom onset [23], we parameterized the transmissibility of disease in asymptomatic, mild symptomatic, and severe symptomatic phases as relative to the pre-symptomatic stage (Additional file 1: Table S1).

### Self-isolation and infection outcomes

SI was implemented 24 h after the onset of symptoms [36]. We assumed that contacts of those who self-isolated were limited to household members with a maximum of three contacts per day, corresponding to the average family size in the province of Ontario [21]. Symptomatic individuals who practiced SI were assumed to stay isolated until the end of their infectious period. We assumed that mild cases recovered without the need for hospitalization, but hospital and intensive care unit (ICU) admissions were included in the model for severe and critically ill individuals. For those who were hospitalized, the average time from symptom onset to admission was uniformly sampled in the range of 2 to 5 days [33]. Hospitalized patients were assumed to be effectively isolated by infection prevention and control measures and were no longer contributing to infection spread. Patients admitted to the hospital occupied a non-ICU bed for an average of 11.5 days before recovering [32, 33]. Further, hospitalized patients had an age-dependent probability of being admitted to ICU, with an average length of 14.4 days stay in ICU [32, 37].

### Model scenarios

We varied the length of SC after implementation from 3 to 16 weeks during the outbreak. We also considered different scenarios for the rates of SI. Of severe cases, 80% would self-isolate within 24 h following symptom onset prior to hospital admission [38]. This assumption is based on prior estimates [39], as well as on the perceived seriousness of COVID-19 infection and emphasis on SI by public health departments. Among COVID-19 cases with mild symptoms, we varied the proportion of SI in the range 0–50%, 24 h after symptom onset. In all scenarios, we calculated the attack rate, cumulative number of ICU admissions, and total ICU bed days throughout the epidemic. We then compared the outcomes measured here when the length of SC and the level of SI among mildly symptomatic cases varied.

### Model implementation

Details of the computational model and its implementation are provided in the Additional file 1. For outbreak scenarios, we calibrated the model to a reproduction number *R*_0_ = 2.5 (as the average number of secondary cases generated by a primary case) to determine the transmission probability per contact in the absence of any control measure [11, 24]. Simulations were seeded with 5 initial symptomatic cases in a 10,000 population, and the results were averaged over 500 independent Monte-Carlo realizations. The start of simulations was set to February 14, 2020, and SC instituted 30 days into the outbreak, corresponding to the start date of March 14, 2020, in Ontario. The model was implemented in Julia language and is available at https://github.com/ABM-Lab/covid19abm.jl.

## Results

In the absence of any control measure, the mean attack rate in the entire population was 60% with *R*_0_ = 2.5. A summary of changes in the attack rate and ICU admissions for scenarios with SI and SC are provided in Additional file 1: Tables S4-S9.

### Effect of SC without SI

**Fig. 1:**
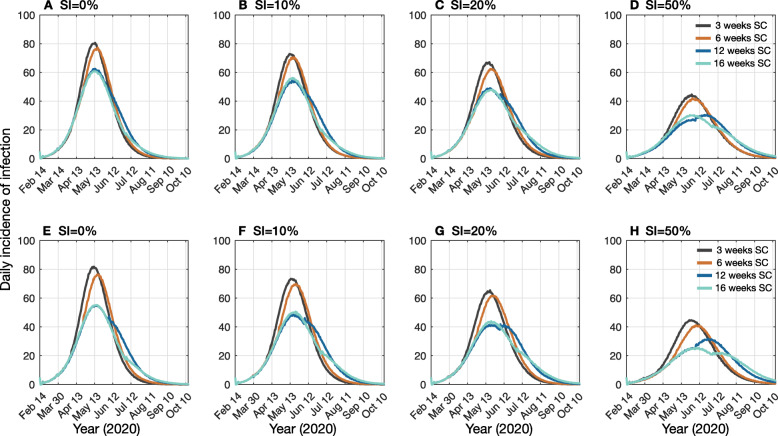
Projected daily incidence of COVID-19 cases per 10,000 populations during outbreaks in Ontario for different proportions of SI, concurrent with SC of varying durations. Top panels (**a**, **b**, **c**, **d**) and bottom panels (**e**, **f**, **g**, **h**) illustrate 60% and 80% reduction of daily contacts among school children aged 5 to 19 years. Color curves correspond to 3 weeks (black), 6 weeks (brown), 12 weeks (blue), and 16 weeks (cyan) duration of SC. *Y*-axis represents the daily incidence of infection and *X*-axis represents time in 30-day increments

**Fig. 2:**
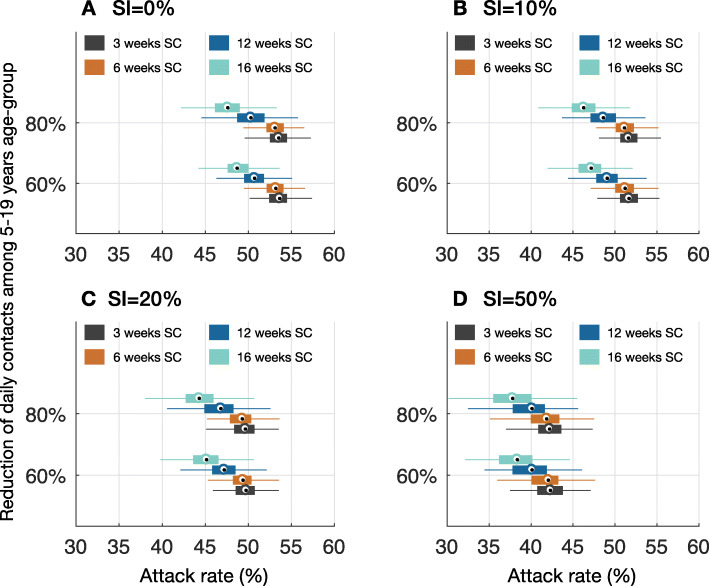
Projected attack rates of COVID-19 per 10,000 population during the outbreak in Ontario for different proportions of SI by symptomatic persons with mild illness, concurrent with SC of varying durations. Color plots correspond to 3 weeks (black), 6 weeks (brown), 12 weeks (blue), and 16 weeks (cyan) duration of SC

**Fig. 3:**
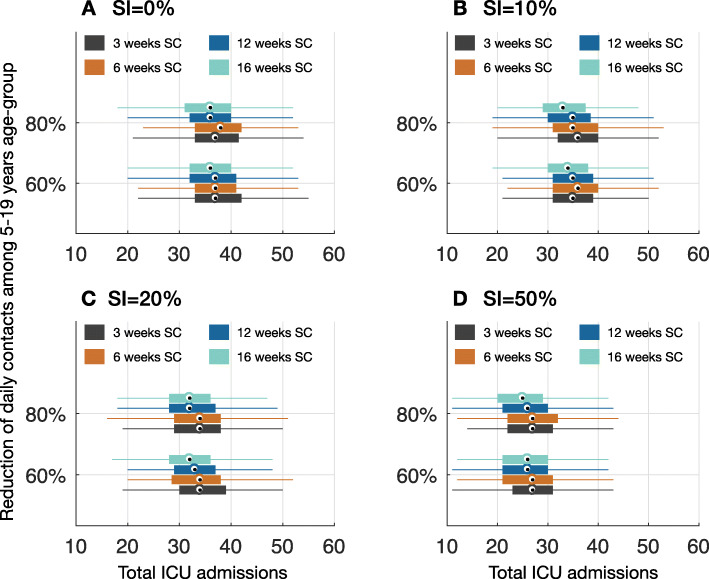
Projected total ICU admissions of COVID-19 patients per 10,000 population during outbreak in Ontario for different proportion of SI by symptomatic persons with mild, concurrent with SC of varying durations. Color bars correspond to 3 weeks (black), 6 weeks (brown), 12 weeks (blue), and 16 weeks (cyan) durations of SC

For the baseline scenario (without SI of mildly symptomatic cases), a 3-week duration of SC in which daily contacts among school children aged 5 to 19 years were reduced by 60%, we projected the COVID-19 outbreak in the province of Ontario to peak in mid-May (Fig. 1a). Extending the length of closure to 16 weeks, essentially to the end of the school year, delayed the peak of outbreak by at most 1 additional week, and reduced the peak of COVID-19 incidence by 24.4% (IQR 23%, 26%) and the overall attack rate by 8.1% (IQR 7.2%, 9.1%) compared to the 3-week SC scenario (Fig. 2a). We projected a reduction of 4.6% (IQR 3.3%, 5.8%) in the total number of ICU admissions during the outbreak with the 16-week SC scenario (Fig. 3a). The total number of ICU bed days per 10,000 population was projected to be 504 (IQR 459, 584) and 482 (IQR 438, 558) in 3-week and 16-week SC scenarios, respectively.

When the reduction of daily contacts among school children was 80% (Fig. 1b), a 16-week SC delayed the peak of outbreak by up to 1 week and reduced the peak of incidence by 32.4% (IQR 31%, 33.6%) compared to the 3-week SC. In this scenario, the reduction in the overall attack rate was increased to 11.8% (IQR 10.8%, 12.7%) (Fig. 2a). The reduction in total ICU admissions throughout the outbreak was 5.5% (IQR 4.3%, 6.7%) (Fig. 3a). We projected the total number of ICU bed days per 10,000 population at 504 (IQR 459, 582) and 476 (IQR 438, 556) when the length of SC was 3 and 16 weeks, respectively.

### Effect of SC with SI

With a 16-week duration of SC, when SI concurrently practiced by 10% of symptomatic individuals with mild illness, the peak of the outbreak was delayed by up to 2 weeks compared to the peak time with a 3-week SC (Fig. 1c, d). Importantly, compared to SC without SI, the peak incidence was lower in all scenarios and reduced further as the length of SC increased. We observed a reduction of 1.3–4.4% in the overall attack rates depending on the length of SC, compared to no SI (Fig. 2b). The reduction of total ICU admissions in the corresponding scenarios varied in the range 3.3–6.6% (Fig. 3b). Increasing the proportion of SI to 20% among mildly symptomatic cases delayed the peak of incidence by an additional 1–4 weeks (Fig. 1e, f), reduced the overall attack rate by 6.3–10.3% (Fig. 2c), and lowered total ICU admissions by 9.1–11.4% compared to no SI (Fig. 3c). We observed a more substantial reduction of attack rates in the range 23.8–25.7% when 50% of mildly symptomatic cases practiced SI (Fig. 2d). Concomitantly, the peak time was projected to occur with a delay of 2 to 6 weeks, depending on the length of SC, compared to no SI. In this scenario, the total ICU admission was reduced by 29.7–32.5%, compared to no SI.

### Effect of epidemiological characteristics

Our results for the limited impact of SC on COVID-19 spread in the absence of SI show a clear contrast to those reported during influenza outbreaks [20, 40]. We therefore investigated the effect of incubation period and the reproduction number as possible explicators (Additional file 1: Fig. S1). The average pre-symptomatic period (as part of the incubation period) is considerably shorter in influenza infection (0.2–1 days) [41, 42] than in COVID-19 (2.3 days) [23]. For a shorter incubation period of 1.5 days, on average [13], simulations indicate that the overall attack rates reduced from 28.5 to 22.2%, when the length of SC increased from 3 to 16 weeks and daily contacts among school children was restricted by 60% (Additional file 1: Table S8). In these scenarios, the cumulative number of ICU admissions over the course of the outbreak was projected to range from 12 to 15 per 10,000 population, depending on the length of SC. When the reduction of daily contacts among school children was 80%, we observed a higher decline in the overall attack rate from 27.8 to 19.7% by increasing the length of SC from 3 to 16 weeks (Additional file 1: Table S8). In this case, the cumulative number of ICU admissions throughout the outbreak reduced from 15 to 11 per 10,000 population. Combined with lower reproduction number of *R*_0_ = 1.5 [14], these results suggest that SC can be substantially more effective during influenza outbreaks, compared to the ongoing COVID-19 outbreaks, even when not augmented by SI.

## Discussion

In the absence of therapeutic antivirals or a vaccine to prevent the spread of SARS-CoV-2, public health and government officials require evidence on appropriate measures to manage population interactions that prevent and slow transmission so that health care systems are not overwhelmed. Research supporting SC for disease outbreaks among other forms of social and physical distancing has been mixed to date, often because of differences in study method and local contexts [5, 43]. Studies found differing outcomes for infection transmission [20, 44, 45] and attack rates [20] depending on the type of study, local conditions, and the nature (duration, scope) of SC. A review of studies in 2014 [43], for example, concluded that “in the absence of evidence to guide practice, public health decision-makers may determine the need to close schools on a case-by-case basis,” taking circumstances of each epidemic, characteristics of the community affected by the epidemic, and other available strategies into account [43]. In contrast to studies deriving data from evidence of decreased transmission during holidays, a recent study measured interactions during reactive SC, demonstrating more than 50% reduction in contact between students [4].

Significantly, several published studies have found that closing schools did not alter contact patterns between school children, family, and older relatives [29, 46, 47]. In our study, we took into account restrictions of social contacts among school children from 60 to 80% during SC [4], without affecting contact patterns between and among other age groups. We found that in the absence of measures to interrupt disease transmission by symptomatic cases, SC has relatively limited impact on outcomes measured here, including attack rates, total number of intensive care unit beds required (Additional file 1: Figs. S2-S4), as well as delay in the peak of outbreaks. However, when SI was implemented, a more substantial reduction of disease burden was achieved, depending on the proportion of cases practicing SI, and the effect of SC was more pronounced in flattening the outbreak and delaying the peak time. This suggests that in the context of COVID-19 infection, rapid case identification and SI by infected individuals, including mildly symptomatic individuals, is of critical importance and a high-impact strategy that can determine the trajectory of outbreaks. This is particularly important in the context of recent findings of high viral loads in infected persons at 0.7 days before symptom onset [23], with potential for significant contribution of silent transmission during the pre-symptomatic stage [48].

While SC has been shown to slow the spread of seasonal influenza in both observational [49, 50] and modeling studies [20, 51], the underlying epidemiologic determinants of these observations are not well understood. The incubation and pre-symptomatic periods, and the reproduction number of SARS-CoV-2 are considerably different from both seasonal and pandemic influenza infections [13, 14]. The incubation period for COVID-19 is estimated at 5.2 days (95% CI: 4.1 to 7.0) [33], which is at least three times longer than the short incubation period of 1.4 days (95% CI 1.3–1.5) for influenza A [13]. Furthermore, the lower bound of estimates for the reproduction number of COVID-19 [11] is comparable to or higher than the upper bound for most estimates of reproduction number of influenza epidemics [14]. While not within the scope of this study, evaluation of the relation between epidemiologic characteristics of infectious diseases and the impact of social distancing measures, and particularly SC, during outbreaks merits further investigation in future studies. The social and economic disruption caused by SC to families and society must of course also be weighed in taking a decision to close schools. Although SC has limited effect on the overall attack rate, it can still reduce attack rates among school children. Thus, alternative measures to reduce contacts among children post-lockdown, such as attendance in shifts, can be considered. Early in the pandemic, it appeared that symptomatic and serious COVID-19 illness in children was uncommon compared to adults [52, 53]. However, recently increasing cases of a serious and sometimes life-threatening syndrome, pediatric COVID-associated multi-inflammatory syndrome (PMIS) have been reported [53]. The etiology of this temporally associated illness is not yet clear. If PMIS is causally related to COVID-19, reduction of pediatric illness by SC may be warranted.

Our findings should be considered in the context of model assumptions and parameters that are based on early estimates and may be subject to uncertainty. As new information and data become available, a better quantification and parameterization of our model could provide more accurate projections on the effectiveness of SC in the presence and absence of SI. Furthermore, the effect of other social distancing measures that have been implemented during the COVID-19 pandemic, such as canceling large public gatherings, having many people work from home, and university closures, is not addressed in the model. Quantifying the effects of these measures in future studies would also provide further insights into control of emerging diseases.

## Conclusion

Our study demonstrates that while SC will mitigate disease transmission during the COVID-19 pandemic when combined by other social distancing measures, it may have markedly lower effectiveness in reducing attack rates and hospitalizations compared to SI. As an important measure of social distancing and in an effort to protect children, SC has been implemented in many countries affected by the COVID-19. However, its effectiveness on curbing the outbreaks has not been investigated. Our findings highlight the importance of epidemiologic parameters of particular infectious diseases on the effectiveness of SC. Importantly, our results show that, in the context of COVID-19 outbreaks, public health measures can be expected to have different effectiveness. For the greatest impact, social distancing measures should be directed at those interventions which are most likely to interrupt the chain of transmission during both pre-symptomatic and symptomatic illness. Interventions such as SI, working at home, social distancing, and mask wearing when moving in the community are expected to reduce the spread of COVID-19 significantly, which would allow the healthcare systems to manage critical care capacity for treatment of severely ill patients within finite resources available.

## Supplementary information

**Additional file 1.** Details of the model and additional simulation results.

## Data Availability

Details of the model are provided in Additional file 1. The computational model is available at https://github.com/ABM-Lab/covid19abm.jl

## Abbreviations

SC: School closure
SI: Self-isolation
ICU: Intensive care unit
COVID-19: Coronavirus disease 2019
PMIS: Pediatric COVID-associated multi-inflammatory syndrome
