# Supplementary material for: Simulating the effect of school closure during COVID-19 outbreaks in Ontario, Canada
Source: BMC Med. 2020 Jul 24;18:230. doi: 10.1186/s12916-020-01705-8 (PMC7378981; doi:10.1186/s12916-020-01705-8)
Supplement: Supplementary file 1 — Additional file 1. Details of the model and additional simulation results. [file 12916_2020_1705_MOESM1_ESM.docx]

**Additional File 1**

**Simulating the effect of school closure during COVID-19 outbreaks in Ontario, Canada**

**Elaheh Abdollahi,^1^ Margaret Haworth-Brockman,^2,3^ Yoav Keynan,^2,4^ Joanne M. Langley,^5^ Seyed M. Moghadas,^1^**

^1^Agent-Based Modelling Laboratory, York University, Toronto, ON M3J 1P3 Canada

^2^National Collaborating Centre for Infectious Diseases, Rady Faculty of Health Sciences, University of Manitoba, Winnipeg, MB R3E 0T5, Canada

^3^Department of Community Health Sciences, and Max Rady College of Medicine, University of Manitoba, Winnipeg MB R3E 0T5, Canada

^4^Department of Medical Microbiology, Max Rady College of Medicine, University of Manitoba, Winnipeg MB R3E 0T5, Canada

^5^Canadian Center for Vaccinology, Dalhousie University, IWK Health Centre and Nova Scotia Health Authority, Halifax, Nova Scotia B3K 6R8 Canada

**Model Structure**

We developed a computational agent-based simulation model for the transmission dynamics of COVID-19. Each agent (representing an individual in a human population) was characterized by time-dependent epidemiological statuses (Fig. S1) of susceptible, infected and incubating, asymptomatic, pre-symptomatic, and symptomatic with either mild, severe, or critical illness, recovered, and dead. We considered hospitalization status for severe and critical illness during which an infected individual is isolated and can no longer transmit the disease.

The duration of stay in each epidemiological status (upon infection) in the model was determined by sampling from available specific distributions, such as the incubation, asymptomatic, pre-symptomatic, duration of communicability after the onset of symptoms, and the length of hospitalization/ICU stays (Table S1). This sampling was done for each individual during the simulations.

The time-unit of the simulation model was one day. The daily number of contacts for agents (with any other agent) was sampled from an age-specific negative-binomial distribution (Table S2) [27], and contacts were distributed among different age groups based on a contact matrix (Table S3) depending on the scenarios of school closure. Disease transmission between susceptible and infected individuals occurred probabilistically at the time of contact, as a result of rejection sampling-based (Bernoulli) trials, where the chance of success is defined by a transmission probability. This transmission probability was determined through a calibration process, so that the average number of secondary cases produced by an infected case (over 500 independent realizations) was the reproduction number of 2.5.


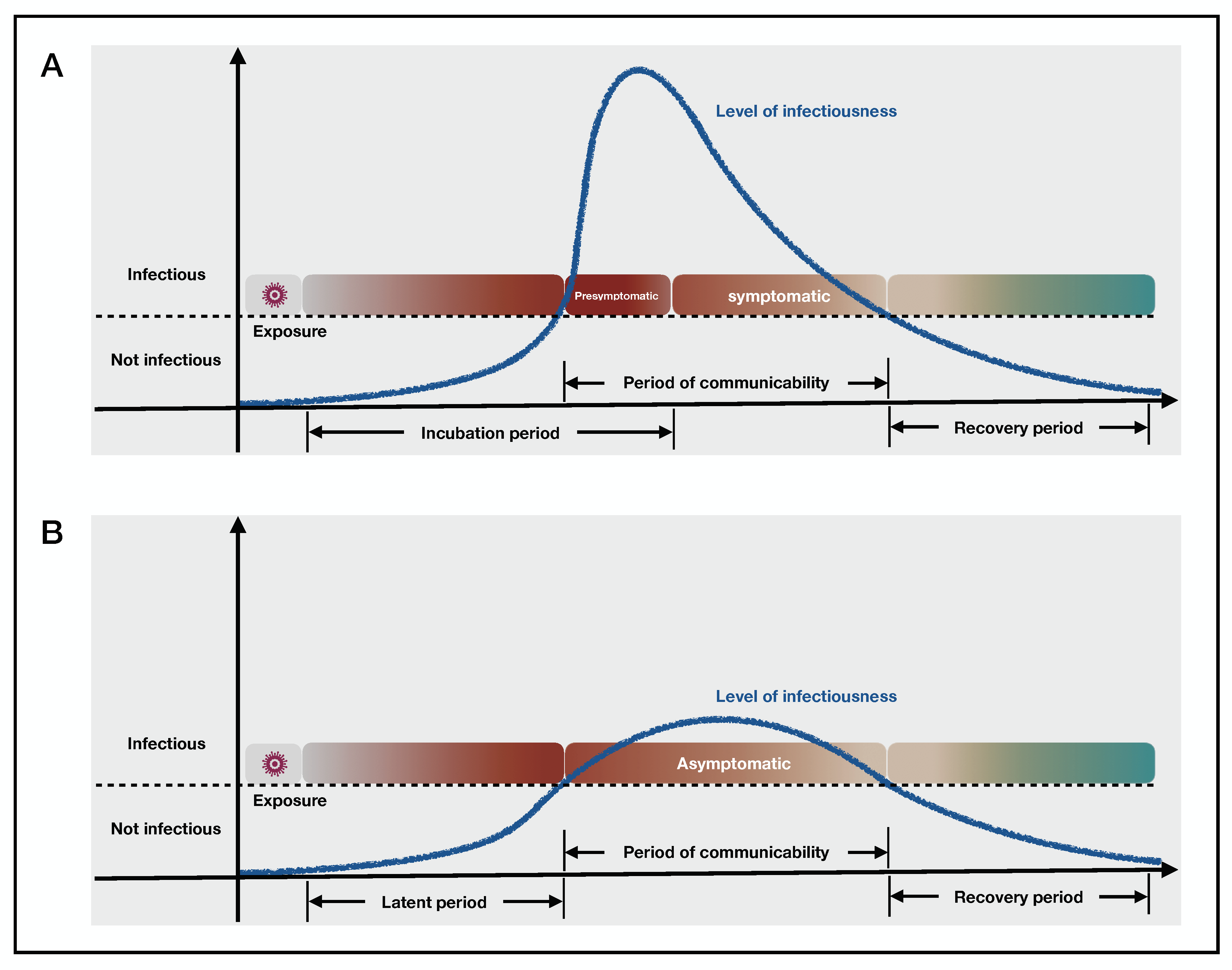


**Figure S1.** Schematic diagram for symptomatic (A) and asymptomatic (B) infections of COVID-19 and the level of infectiousness in different stages of the disease.

**Table S1.** Description of parameters and their values/ranges for each age group

| **Description** | **0-4** | **5-19** | **20-49** | **50-64** | **65+** | **Reference** |
| --- | --- | --- | --- | --- | --- | --- |
| Transmission probability per contact during pre-symptomatic stage | 0.041 | | | | | Calibrated to R_0_=2.5  [11, 24 |
| Relative infectivity of severe symptomatic compared to pre-symptomatic | 0.89 | | | | | [30-32] |
| Relative infectivity of mild symptomatic compared to pre-symptomatic | 0.44 | | | | | [23,31] |
| Relative infectivity of asymptomatic compared to pre-symptomatic | 0.11 | | | | | [31] |
| incubation period (days) | LogN(5.2, 0.1) | | | | | [11,12] |
| Proportion of infections that is asymptomatic | 0.25 | 0.25 | 0.14 | 0.07 | 0.07 | Assumed |
| Proportion of cases that exhibit mild symptoms | 0.95 | 0.9 | 0.85 | 0.60 | 0.20 | [30,32] |
| Proportion of severe symptomatic cases who practice self-isolation upon symptom onset | 0.8 | | | | | Assumed |
| Pre-symptomatic period (days) | Gamma (1.058, 2.174) | | | | | Derived from [23] |
| Infectious period from onset of symptoms | Gamma (2.768, 1.1563) | | | | | Estimated from [34] |
| Asymptomatic period (days) | Gamma (5, 1) | | | | | Derived from  [31] |
| Probability of self-isolation 24 hours after symptom onset for mild cases | 0 – 0.5 | 0 – 0.5 | 0 – 0.5 | 0 – 0.5 | 0 – 0.5 | Varied |
| Time from symptom onset to self-isolation (days) | 1 | | | | | Assumed |
| Time from symptom onset to hospitalization (days) | Unif(2,5) | | | | | [33] |
| Proportion of symptomatic patients with severe and critical illness requiring hospital care or ICU | Unif(0.02,0.03) | Unif(0.02,0.03) | Unif(0.28,0.34) | Unif(0.28,0.34) | Unif(0.60,0.68) | Estimated from [22,35] |
| Proportion of hospitalized cases requiring ICU | Unif(0.01,0.015) | Unif(0.01,0.015) | Unif(0.03,0.05) | Unif(0.05, 0.2) | Unif(0.05,0.15) | Estimated from [22] |
| Length of hospital stay before recovery (days) | Gamma(4.5, 2.75) truncated between 8 and 17 | | | | | [33] |
| Length of ICU stay before recovery (days) | Gamma(5.26, 2.74) truncated between 10 and 19 | | | | | [33] |

**Table S2.** Mean and standard deviation of the negative binomial distributions for the daily number of contacts in different age groups.

| **Age group** | **Mean (standard deviation) of daily number of contacts** |
| --- | --- |
| 0-4 | 10.21 (7.65) |
| 5-19 | 16.79 (11.72) |
| 20-49 | 13.79 (10.50) |
| 50-64 | 11.26 (9.59) |
| 65+ | 8.00 (6.96) |

**Table S3.** Contact matrices when school is open and for school closure with 60% and 80% reduction of contacts among school children.

1. Regular contacts

| School open | 0-4 | 5-19 | 20-49 | 50-64 | 65+ |
| --- | --- | --- | --- | --- | --- |
| 0-4 | 0.2287 | 0.1839 | 0.4219 | 0.1116 | 0.0539 |
| 5-19 | 0.0276 | 0.5964 | 0.2878 | 0.0591 | 0.0291 |
| 20-49 | 0.0376 | 0.1454 | 0.6253 | 0.1423 | 0.0494 |
| 50-64 | 0.0242 | 0.1094 | 0.4867 | 0.2723 | 0.1074 |
| 65+ | 0.0207 | 0.1083 | 0.4071 | 0.2193 | 0.2446 |

1. 60% reduced contacts among 5-19 years age-group

| School closed | 0-4 | 5-19 | 20-49 | 50-64 | 65+ |
| --- | --- | --- | --- | --- | --- |
| 0-4 | 0.2287 | 0.1839 | 0.4219 | 0.1116 | 0.0539 |
| 5-19 | 0.0276 | 0.2386 | 0.2878 | 0.0591 | 0.0291 |
| 20-49 | 0.0376 | 0.1454 | 0.6253 | 0.1423 | 0.0494 |
| 50-64 | 0.0242 | 0.1094 | 0.4867 | 0.2723 | 0.1074 |
| 65+ | 0.0207 | 0.1083 | 0.4071 | 0.2193 | 0.2446 |

1. 80% reduced contacts among 5-19 years age-group

| School closed | 0-4 | 5-19 | 20-49 | 50-64 | 65+ |
| --- | --- | --- | --- | --- | --- |
| 0-4 | 0.2287 | 0.1839 | 0.4219 | 0.1116 | 0.0539 |
| 5-19 | 0.0276 | 0.1193 | 0.2878 | 0.0591 | 0.0291 |
| 20-49 | 0.0376 | 0.1454 | 0.6253 | 0.1423 | 0.0494 |
| 50-64 | 0.0242 | 0.1094 | 0.4867 | 0.2723 | 0.1074 |
| 65+ | 0.0207 | 0.1083 | 0.4071 | 0.2193 | 0.2446 |

**Results for reduction of attack rate and ICU admissions during school closure with 60% reduction of daily contacts among school children**

**Table S4.** Projected median attack rates and interquartile range in different scenarios for length of SC and proportion of SI.

| 60% reduction of daily contacts among school children | Projected median attack rates (IQR) | | | |
| --- | --- | --- | --- | --- |
|  | Length of SC in weeks | | | |
| Proportion of SI | 3 | 6 | 12 | 16 |
| 0% | 53.6%  (52.4%, 54.5%) | 53.2%  (52.1%, 54.1%) | 50.7%  (49.5%, 51.8%) | 48.7%  (47.6%, 50.0%) |
| 10% | 51.7%  (50.6%, 52.7%) | 51.2%  (50.0%, 52.3%) | 49.1%  (47.8%, 50.3%) | 47.2%  (47.7%, 48.3%) |
| 20% | 49.7%  (48.5%, 50.7%) | 49.4%  (48.2%, 50.3%) | 47.2%  (45.8%, 48.5%) | 45.2%  (43.6%, 46.5%) |
| 50% | 42.3%  (40.7%, 43.8%) | 42.1%  (40.1%, 43.3%) | 40.1%  (37.8%, 41.9%) | 38.4%  (36.2%, 40.1%) |

**Table S5.** Projected median ICU admissions and interquartile range in different scenarios for length of SC and proportion of SI.

| 60% reduction of daily contacts among school children | Projected median of total ICU admissions (IQR) | | | |
| --- | --- | --- | --- | --- |
|  | Length of SC in weeks | | | |
| Proportion of SI | 3 | 6 | 12 | 16 |
| 0% | 37  (33, 42) | 37  (33, 41) | 37  (32, 41) | 36  (32, 40) |
| 10% | 35  (31, 39) | 36  (31, 40) | 35  (31, 39) | 34  (30, 38) |
| 20% | 34  (30, 39) | 34  (29, 38) | 33  (29, 37) | 32  (28, 36) |
| 50% | 27  (23, 31) | 27  (21, 31) | 26  (21, 30) | 26  (21, 30) |

**Results for reduction of attack rate and ICU admissions during school closure with 80% reduction of daily contacts among school children**

**Table S6.** Projected median attack rates and interquartile range in different scenarios for length of SC and proportion of SI.

| 80% reduction of daily contacts among school children | Projected median attack rates (IQR) | | | |
| --- | --- | --- | --- | --- |
|  | Length of SC in weeks | | | |
| Proportion of SI | 3 | 6 | 12 | 16 |
| 0% | 53.5%  (52.5%, 54.5%) | 53.1%  (52.1%, 54.1%) | 50.3%  (48.7%, 51.9%) | 47.6%  (46.1%, 40.0%) |
| 10% | 51.6%  (60.6%, 52.7%) | 51.1%  (50.1%, 52.3%) | 48.6%  (47.1%, 50.1%) | 46.3%  (44.9%, 47.7%) |
| 20% | 49.7%  (48.4%, 50.7%) | 49.3%  (47.9%, 50.3%) | 46.8%  (44.9%, 48.3%) | 44.3%  (42.7%, 45.9%) |
| 50% | 42.2%  (40.8%, 43.6%) | 42.1%  (40.0%, 43.4%) | 40.1%  (37.8%, 41.6%) | 37.8%  (35.5%, 40.1%) |

**Table S7.** Projected median ICU admissions and interquartile range in different scenarios for length of SC and proportion of SI.

| 80% reduction of daily contacts among school children | Projected median of total ICU admissions (IQR) | | | |
| --- | --- | --- | --- | --- |
|  | Length of SC in weeks | | | |
| Proportion of SI | 3 | 6 | 12 | 16 |
| 0% | 37  (33, 41) | 38  (33, 42) | 36  (32, 40) | 36  (31, 40) |
| 10% | 36  (32, 40) | 35  (31, 40) | 35  (30, 39) | 33  (29, 38) |
| 20% | 34  (29, 38) | 34  (29, 38) | 32  (28, 37) | 32  (28, 37) |
| 50% | 27  (22, 31) | 27  (22, 32) | 26  (21, 30) | 25  (20, 29) |

**Table S8.** Age-specific attack rates with 60% reduction of daily contacts among school children. Attack rates were calculated by dividing the total number of infections in each age group by the total population of the same age group.

| **Level of SI: 0%** | Projected median attack rates for different age groups | | | | |
| --- | --- | --- | --- | --- | --- |
| **Length of SC in weeks** | **0-4** | **5-19** | **20-49** | **50-64** | **65+** |
| 3 | 36% | 67% | 63% | 41% | 24% |
| 6 | 36% | 66% | 63% | 41% | 24% |
| 12 | 36% | 58% | 62% | 41% | 24% |
| 16 | 34% | 51% | 60% | 39% | 23% |
| **Level of SI: 10%** |  |  |  |  |  |
| 3 | 34% | 64% | 60% | 39% | 23% |
| 6 | 34% | 63% | 60% | 39% | 23% |
| 12 | 33% | 55% | 59% | 38% | 22% |
| 16 | 33% | 50% | 59% | 38% | 22% |
| **Level of SI: 20%** |  |  |  |  |  |
| 3 | 32% | 62% | 57% | 37% | 22% |
| 6 | 32% | 60% | 57% | 36% | 21% |
| 12 | 31% | 54% | 57% | 36% | 21% |
| 16 | 31% | 49% | 56% | 36% | 21% |
| **Level of SI: 50%** |  |  |  |  |  |
| 3 | 25% | 52% | 48% | 29% | 17% |
| 6 | 24% | 49% | 46% | 28% | 16% |
| 12 | 23% | 43% | 44% | 28% | 16% |
| 16 | 23% | 41% | 45% | 28% | 16% |

**Table S9.** Age-specific attack rates with 80% reduction of daily contacts among school children. Attack rates were calculated by dividing the total number of infections in each age group by the total population of the same age group.

| **Level of SI: 0%** | Projected median attack rates for different age groups | | | | |
| --- | --- | --- | --- | --- | --- |
| **Length of SC in weeks** | **0-4** | **5-19** | **20-49** | **50-64** | **65+** |
| 3 | 36% | 67% | 63% | 41% | 24% |
| 6 | 36% | 65% | 62% | 41% | 24% |
| 12 | 35% | 55% | 61% | 40% | 23% |
| 16 | 33% | 46% | 50% | 38% | 23% |
| **Level of SI: 10%** |  |  |  |  |  |
| 3 | 34% | 65% | 60% | 39% | 23% |
| 6 | 34% | 64% | 60% | 38% | 23% |
| 12 | 32% | 54% | 58% | 38% | 22% |
| 16 | 32% | 47% | 58% | 37% | 22% |
| **Level of SI: 20%** |  |  |  |  |  |
| 3 | 32% | 61% | 57% | 36% | 21% |
| 6 | 31% | 60% | 56% | 36% | 21% |
| 12 | 30% | 51% | 55% | 35% | 20% |
| 16 | 30% | 46% | 54% | 35% | 20% |
| **Level of SI: 50%** |  |  |  |  |  |
| 3 | 25% | 52% | 48% | 29% | 17% |
| 6 | 24% | 48% | 45% | 28% | 16% |
| 12 | 23% | 45% | 45% | 28% | 16% |
| 16 | 23% | 39% | 44% | 27% | 15% |

**Results for prevalence of ICU beds utilization**

**Figure S2.** Projected prevalence of ICU beds utilization for age group 20-49 during outbreaks in Ontario for different proportions of SI, concurrent with SC of varying durations. Top panels (A, B, C, D) and bottom panels (E, F, G, H) illustrate 60% and 80% reduction of daily contacts among school children aged 5 to 19 years. Colour curves correspond to 3 weeks (black), 6 weeks (brown), 12 weeks (blue), and 16 weeks (cyan) duration of SC. Y-axis represents the daily incidence of infection and X-axis represents time in 30-day increments.

**Figure S3.** Projected prevalence of ICU beds utilization for age group 50-65 during outbreaks in Ontario for different proportions of SI, concurrent with SC of varying durations. Top panels (A, B, C, D) and bottom panels (E, F, G, H) illustrate 60% and 80% reduction of daily contacts among school children aged 5 to 19 years. Colour curves correspond to 3 weeks (black), 6 weeks (brown), 12 weeks (blue), and 16 weeks (cyan) duration of SC. Y-axis represents the daily incidence of infection and X-axis represents time in 30-day increments.

**Figure S4.** Projected prevalence of ICU beds utilization for age group 65+ during outbreaks in Ontario for different proportions of SI, concurrent with SC of varying durations. Top panels (A, B, C, D) and bottom panels (E, F, G, H) illustrate 60% and 80% reduction of daily contacts among school children aged 5 to 19 years. Colour curves correspond to 3 weeks (black), 6 weeks (brown), 12 weeks (blue), and 16 weeks (cyan) duration of SC. Y-axis represents the daily incidence of infection and X-axis represents time in 30-day increments.

**Results for *R*_0_=1.5 and the average incubation period of 1.5 days**

We calibrated the model to *R*_0_=1.5 (attack rate of 33%) and determined the transmission probability per contact when the incubation period was sampled from a Log-Normal distribution with the mean of 1.5 days. We assumed a pre-symptomatic period of 1 day estimated for children. This period is shorter (0.2 days) for adults [41,42]. We then ran simulations when school closure was implemented on 30 after the start of the outbreak, in the absence of self-isolation. The length of school closure was the same as the scenarios described in the main text for 3, 6, 12 and 16 weeks. Simulations for daily incidence of infection are shown in Figure S5. Summary of outcomes measured are reported in Table S10.

**Table S10.** Projected median attack rates and cumulative ICU admissions per 10,000 population for different lengths of school closure (in the absence of SI), with 60% and 80% reduction of daily contacts among school children.

|  | Reduction of daily contacts among school children | | | |
| --- | --- | --- | --- | --- |
|  | 60% | | 80% | |
| Length of school closure (weeks) | Attack rate | ICU admissions | Attack rate | ICU admissions |
| 3 | 28.5% | 15 | 27.8% | 15 |
| 6 | 27.3% | 15 | 27.2% | 15 |
| 12 | 24.3% | 14 | 22.9% | 13 |
| 16 | 22.2% | 12 | 19.7% | 11 |

**Figure S5.** Projected daily incidence of infection during outbreaks in Ontario for different lengths of school closure. Panels (A) and (B) correspond to 60% and 80% reduction of daily contacts among school children aged 5 to 19 years, respectively. Colour curves correspond to 3 weeks (black), 6 weeks (brown), 12 weeks (blue), and 16 weeks SC (cyan). Y-axis represents the daily incidence of infection and X-axis represents time in 30-day increments.
